# A Honeycomb‐Structured CoF_2_‐Modified Separator Enabling High‐Performance Lithium−Sulfur Batteries

**DOI:** 10.1002/smsc.202300006

**Published:** 2023-04-06

**Authors:** Wenxin Liu, Yuhang Chu, Jinwei Zhou, Xuanfeng Chen, Yujie Wang, Jinhui Li, Feixiang Wu

**Affiliations:** ^1^ Faculty of Materials Metallurgy and Chemistry Jiangxi University of Science and Technology Ganzhou 341000 China; ^2^ School of Metallurgy and Environment Central South University Changsha 410083 China

**Keywords:** cobalt fluoride, lithium–sulfur batteries, modified separators, polysulfide shuttle, sulfur cathodes

## Abstract

Sulfur cathode materials in lithium–sulfur chemistry suffer from poor electronic conductivity and shuttle of lithium polysulfides during charging and discharging. Serious shuttle effects and the sluggish redox reaction kinetics of polysulfides severely limit the development of lithium–sulfur batteries with high sulfur loading, impeding the practical process of lithium–sulfur batteries. Herein, a honeycomb73x02010;structured CoF_2_@C is introduced as a functional layer adhered to the separator, achieving rapid lithium‐ion transport, high catalytic activity, and suppressed shuttle effect simultaneously. As a result, the cell with CoF_2_‐modified separator presents satisfactory cycle stability with a capacity decay of 0.076% per cycle within 300 cycles at 1 C rate with the sulfur loading of 2.0 mg cm^−2^. A low‐capacity decay of 0.088% per cycle for 200 cycles at 0.2 C is also achieved with sulfur loading of 3.0 mg cm^−2^. In addition, a high‐capacity retention of 697.5 mA g^−1^ is achieved with sulfur loading of 4.0 mg cm^−2^ and the electrolyte volume/sulfur mass (E/S) ratio of 8 μL mg^−1^.

## Graphical abstract

The separator is modified by the honeycomb structured CoF_2_@C, which can effectively adsorb polysulfide, catalyze Li–S conversion, and reduce voltage hysteresis, achieving high‐performance Li–S batteries. During operation, the produced CoF_2_@C demonstrates great stability on morphology and phase after long‐term cycling.

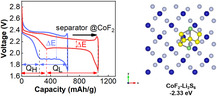

## 1 Introduction

Traditional lithium‐ion batteries (LIBs) are reaching their bottlenecks due to the theoretical capacity density limitations, which cannot meet the growing demands for electric vehicles and mobile power devices.^[^ ^1^, ^2^, ^3^, ^4^ ^]^ Lithium–sulfur batteries have been prioritized as the alternative for the development of next‐generation high‐energy energy‐storage systems, attributed to the low cost, abundant supply, and environmental friendliness of sulfur, as well as its extremely high theoretical specific capacity of 1675 mAh g^−1^ and theoretical energy density of 2800 Wh kg^−1^.^[^ ^5^, ^6^, ^7^, ^8^, ^9^, ^10^ ^]^ However, a series of issues remain to be solved, such as poor conductivity of sulfur materials, shuttle effect of dissolved lithium polysulfide, and structural destruction of the cathode material caused by volume changes during the cycle, which would result in dramatical capacity decay, low Coulomb efficiency, and poor rate performance of lithium–sulfur batteries.^[^ ^11^, ^12^, ^13^, ^14^, ^15^ ^]^

During the past decades, numerous efforts have been made to design and modify the functional sulfur cathode materials. Typically, carbon‐based materials, metal oxides, metal sulfides, and metal nitrides are introduced as the sulfur host materials to enable high electrochemical performance of Li–S batteries.^[^ ^16^, ^17^, ^18^, ^19^, ^20^, ^21^, ^22^ ^]^ The sulfur–carbon composite can effectively inhibit the shuttle effect via the physical adsorption of lithium polysulfide and greatly reduce the electrochemical impedance of the battery, ascribed to the good conductivity and high porosity of the carbon material itself.^[^ ^23^, ^24^, ^25^ ^]^ Additionally, polar compounds, such as metal oxides, metal fluorides, and metal sulfides, have been developed to enhance the chemical anchoring of lithium polysulfides and promote its redox kinetics during cycling, hence improving the usage of active ingredients.^[^ ^26^, ^27^, ^28^, ^29^, ^30^, ^31^, ^32^ ^]^ However, the strategy of using the host materials usually would lead to the limited sulfur loading, excessive electrolyte usage, and high manufacturing cost. Recently, the functional separator and carbon interlayer separator have been considered as highly effective methods to suppress the diffusion of soluble lithium polysulfides.^[^ ^33^, ^34^ ^]^ First, the functional layer is used as a physical barrier to hinder the shuttle effect of polysulfides. Furthermore, the designed functional layer can adsorb the soluble polysulfide during the discharge and charge process so that its capacity can be reused, and some functional layers can also catalyze the conversion of polysulfide and improve its redox kinetics. For example, Su et al. reported an insertion of the electrolyte‐permeable microporous carbon paper (MCP) between the separator and cathode.^[^ ^35^ ^]^ This design of cell can effectively decrease the resistance of cathodes, resulting in an enhancement of active material utilization. Liu et al. reported a nano‐SiO_2_ blending polyetherimide separator modified with acetylene black/poly(vinylpyrrolidone) coating layer.^[^ ^36^ ^]^ The produced coating layer demonstrated excellent adsorption capacity on polysulfides and accelerated redox reaction among polysulfides. Xiao et al proposed that coating the surface of a C–S cathode with a graphene/TiO_2_ film traps and suppresses the dissolution of polysulfides, alleviating the undesirable shuttle effect.^[^ ^37^ ^]^ Ma et al. reported that the separators modified with polypyrrole nanotubes, polypyrrole nanowires, and reduced graphene oxide, respectively, were used for Li–S batteries.^[^ ^38^ ^]^ The results provided that all the conductive materials for the separator surface decoration inhibited the migration of lithium polysulfides in the electrolyte and decreased the polarization of sulfur cathodes. For this reason, designing a functional layer between the separator and cathode is promising to achieve polysulfide adsorption and catalysis.

Herein, we develop a functional separator modified by the honeycomb structured CoF_2_@C for lithium–sulfur batteries. The advantages of the functional separator are as follows. 1) The 3D channels of conductive honeycomb‐structured carbon substrate facilitate more uniform and fast lithium‐ion transport during charging and discharging. 2) CoF_2_ embedded on the surface can chemically trap the soluble lithium polysulfide via Lewis acid–base interaction, thus confining lithium polysulfide to the side of the cathode. Moreover, the CoF_2_ nanoparticles can simultaneously exhibit the electrocatalytic effect and enhance the conversion kinetics of lithium polysulfide. 3) There is so little CoF_2_ to modify separator, which greatly reduces the impact on the mass energy density of the battery. In addition, using graphite and sulfur composites as working electrodes reduces costs and meets the demands of high‐surface‐loading standards for industrial production. As a result, the honeycomb structured CoF_2_@C as a functional layer allows for a high initial capacity of 899.5 mA g^−1^ at 0.2 C with the sulfur loading of 3.0 mg cm^−2^ and excellent cycle performance with a capacity fading of 0.076% per cycle for 300 cycles at 1 C. In addition, a high‐capacity retention of 697.5 mA g^−1^ is also achieved with the sulfur loading of 4.0 mg cm^−2^ and the electrolyte volume/sulfur mass (E/S) ratio of 8 μL mg^−1^.

## 2 Results and Discussion

**Figure 1:**
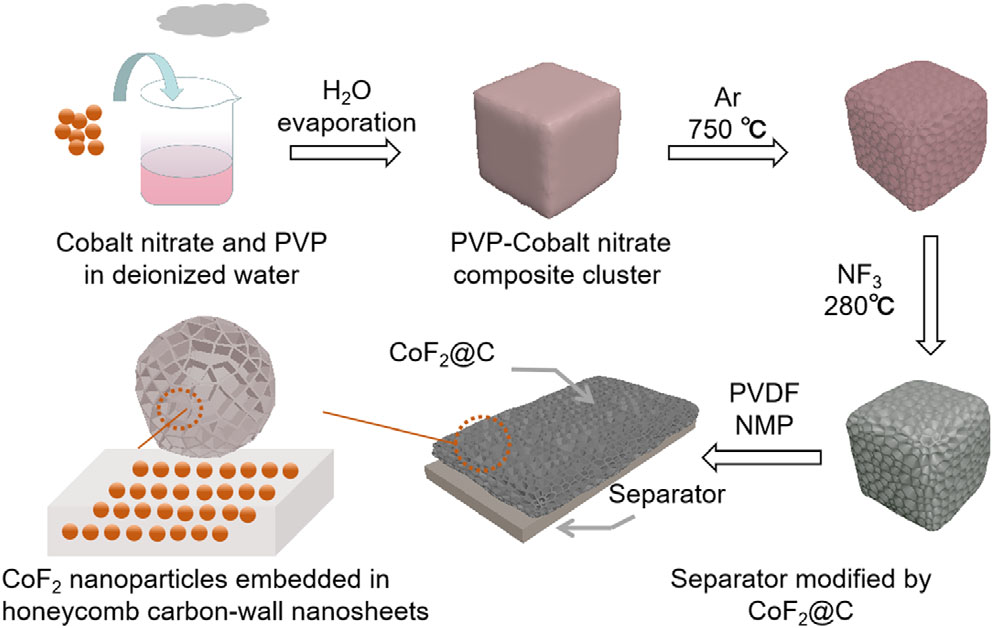
Schematic diagram of the synthesis of honeycomb structured CoF_2_@C‐modified separator.

**Figure** **1** reveals the synthesis route of the honeycomb structured CoF_2_@C composite. The polyvinylpyrrolidone (PVP) and the cobalt nitrate with the mass ratio of 1:1 were introduced to form a gel after the mixed solution was completely dried under 90 °C. Subsequently, the carbonization and fluorination were carried out. As a result, the CoF_2_ nanoparticles homogeneously embedded in the conductive honeycomb structured carbon matrixes (CoF_2_@C) were obtained. Finally, the honeycomb structured CoF_2_@C was dispersed in the separator by the coating process method.

**Figure 2:**
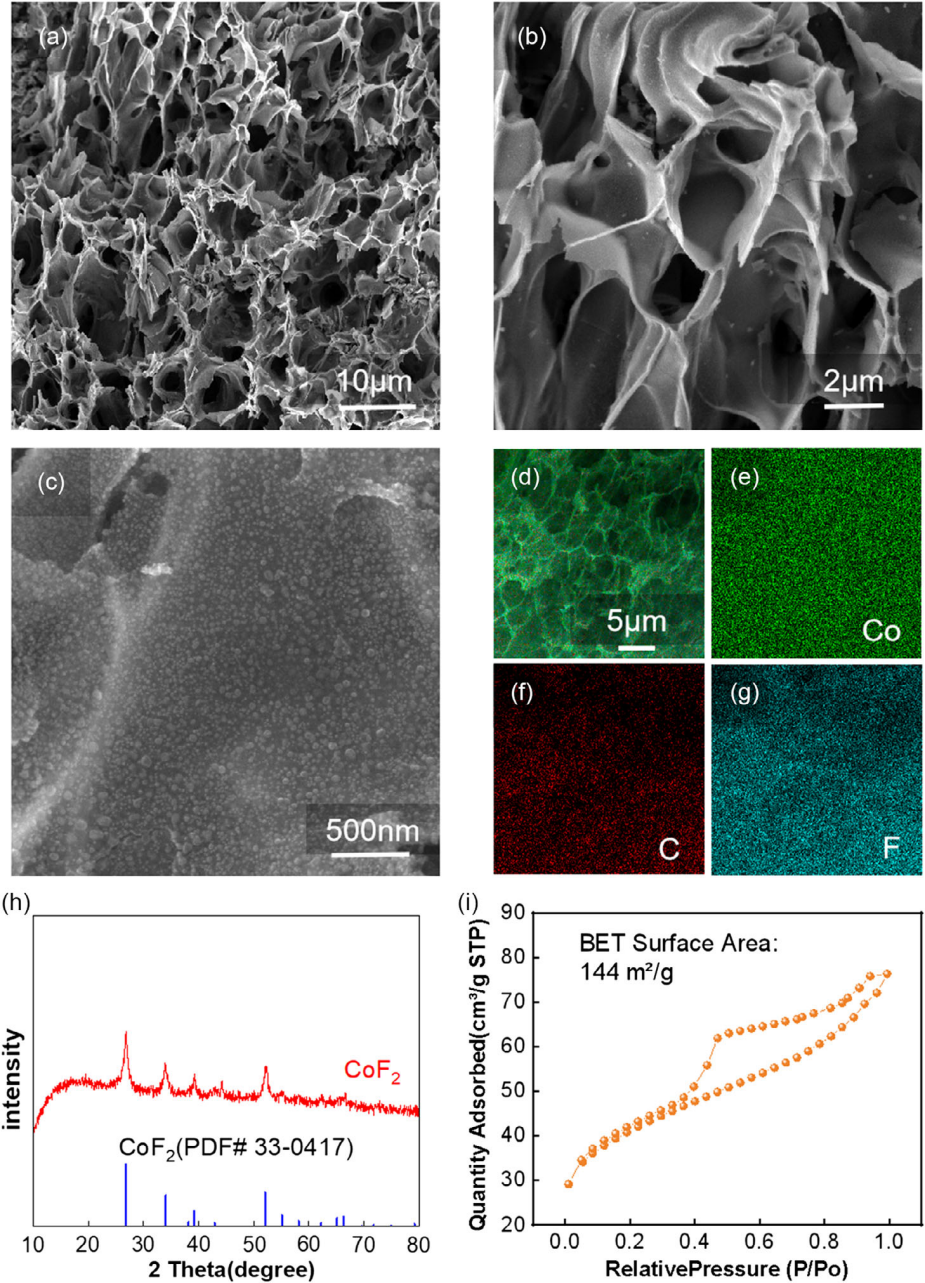
a–c) SEM images of CoF_2_@C. d–g) Elemental mapping of C, Co, and F in CoF_2_. h) XRD pattern of CoF_2_. i) BET surface area of the CoF_2_@C.

The morphology of the honeycomb structured CoF_2_@C was observed by scanning electron microscopy (SEM). **Figure** **2**a‐g shows the morphology and element distribution of CoF_2_@C, and the CoF_2_ nanoparticles are uniformly distributed across the honeycomb structure carbon surface with a side length of about 2 μm. Figure 2h demonstrates the X‐ray diffraction (XRD) patterns of the final product CoF_2_@C. CoF_2_ phase is verified by the four typical peaks at 26.7°, 34.0°, 39.1°, and 52.0°, which agree with PDF card of CoF_2_ (no. 33‐0417). In the Brunauer–Emmett–Teller (BET) specific surface area test (as shown in Figure 2i), the honeycomb structured CoF_2_@C powder provides a high specific surface area of 144 m^2^ g^−1^. These are multiple honeycomb channels which are favorable for lithium polysulfides adsorption in the Li–S battery. The powder was weighed after carbonization and fluorination, and this weight difference was used to calculate the fluorine content, demonstrating 82.5 wt% of CoF_2_ in the composite material.

**Figure 3:**
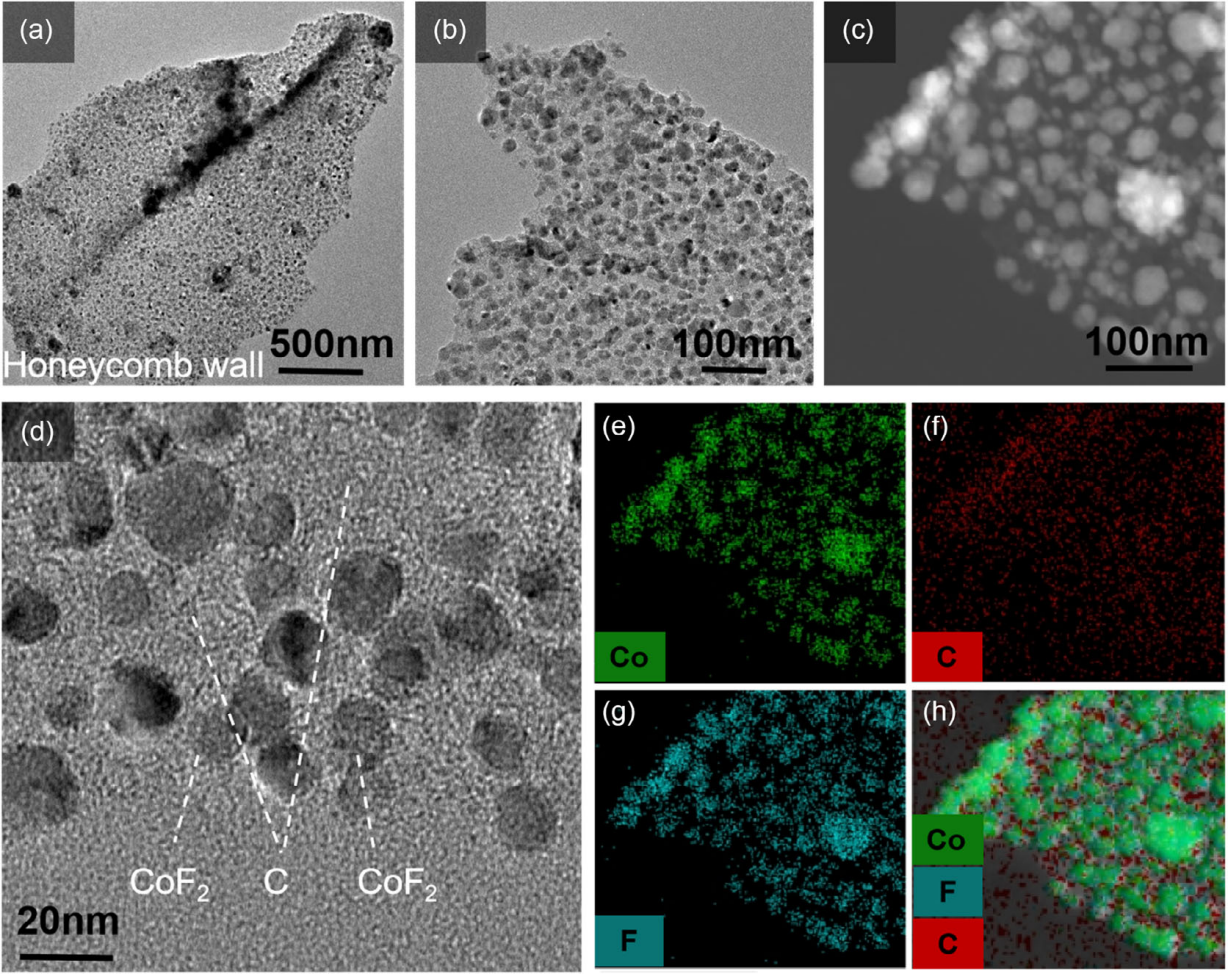
STEM studies of as‐produced honeycomb CoF_2_@C composite. a,b) BF‐STEM and d) HRTEM images of CoF_2_@C composite; c) HAADF‐STEM image of CoF_2_@C composite; EDS images: e) cobalt; f) carbon; g) fluorine; h) their overlapped maps, showing isolated CoF_2_ nanoparticles embedded in honeycomb carbon‐walled nanosheets.

In order to explore more details of the CoF_2_@C composite, high‐resolution transmission electron microscope (HRTEM) studies were carried out on the sample to study the morphology, size, shape, and distribution of the nanoparticles. The (BF)‐ and high‐angle annular dark field (HAADF)‐scanning transmission electron microscopy (STEM) images exhibit a projection of the honeycomb structure. The BF‐, HAADF‐STEM, and HRTEM images reveal that the honeycomb wall consists of carbon nanosheets embedding isolated CoF_2_ that is sphere‐like nanoparticles with size of 10–20 nm (**Figure** **3**a,b,d). The HAADF‐STEM image combined with energy‐dispersive spectrometry (EDS) elemental maps (Figure 3e–h) show the Co, C, and F distribution within the CoF_2_@C composite, further proving that CoF_2_ nanoparticles are uniformly embedded in the carbon matrix.

**Figure 4:**
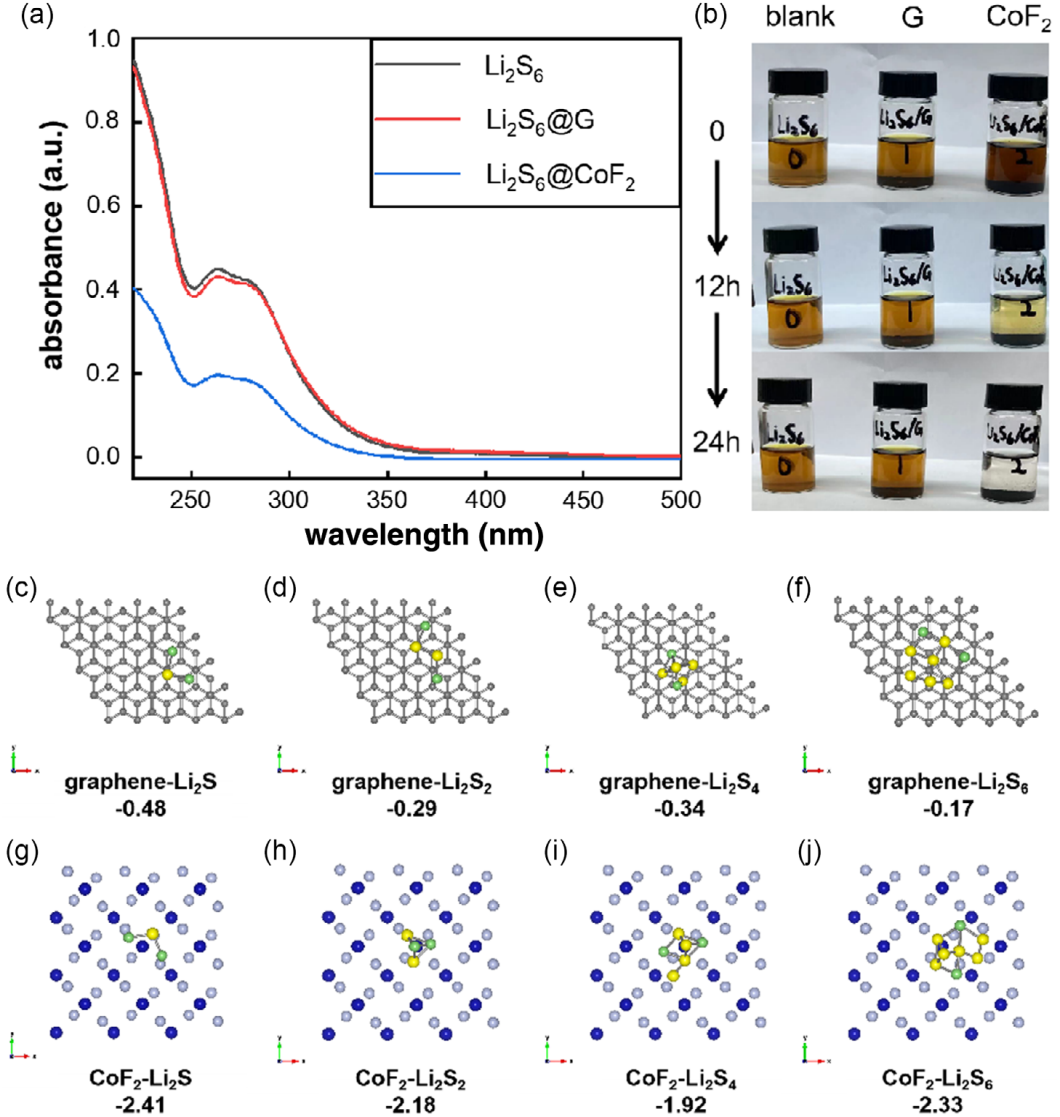
a) UV–vis adsorption spectra of Li_2_S_6_ solution on different materials. b) Photos of Li_2_S_6_ solution before and after contacting graphite or CoF_2_@C for 24 h. c–f) Binding geometric models and binding energy between the G and Li_2_S_*X*_ (1 ≤ *x* ≤ 6). g–j) Binding geometric models and binding energy between the CoF_2_ and Li_2_S_X_ (1 ≤ *x* ≤ 6).

Polysulfides adsorption experiments were conducted to investigate the interaction between honeycomb CoF_2_@C and lithium polysulfides. Honeycomb structured CoF_2_@C and G (graphite) are physically mixed with Li_2_S_6_ in DOL/ DME solution for 24 h, after which the liquid supernatant is collected for further examination. From the optical images (as shown in **Figure** **4**b), it is demonstrated that the color of Li_2_S_6_ solution could be obviously changed from brown into colorless, which indicates that lithium polysulfides are sufficiently absorbed by honeycomb CoF_2_@C after 24 h. By UV spectral analysis, the intensity of the characteristic absorption peak of Li_2_S_6_ would be the weakest after lithium polysulfide interacts with honeycomb CoF_2_. This means that honeycomb structure CoF_2_@C has a strong adsorption effect on lithium polysulfide. The binding geometric models and binding energy between Li_2_S_*X*_ (1bindinand CoF_2_@C or G were studied by density functional theory (DFT) calculations. As shown in Figure 4c–j, CoF_2_ provides stronger adsorption capacity on LiPSs with the binding energies of −2.41, −2.18, −1.92, and −2.33 eV than graphite with the binding energies of −0.48, −0.29, −0.34, and −0.17 eV, respectively. This result is consistent with the conclusion of the visual adsorption experiment in Figure 4b and the corresponding UV/vis adsorption spectra in Figure 4a.

**Figure 5:**
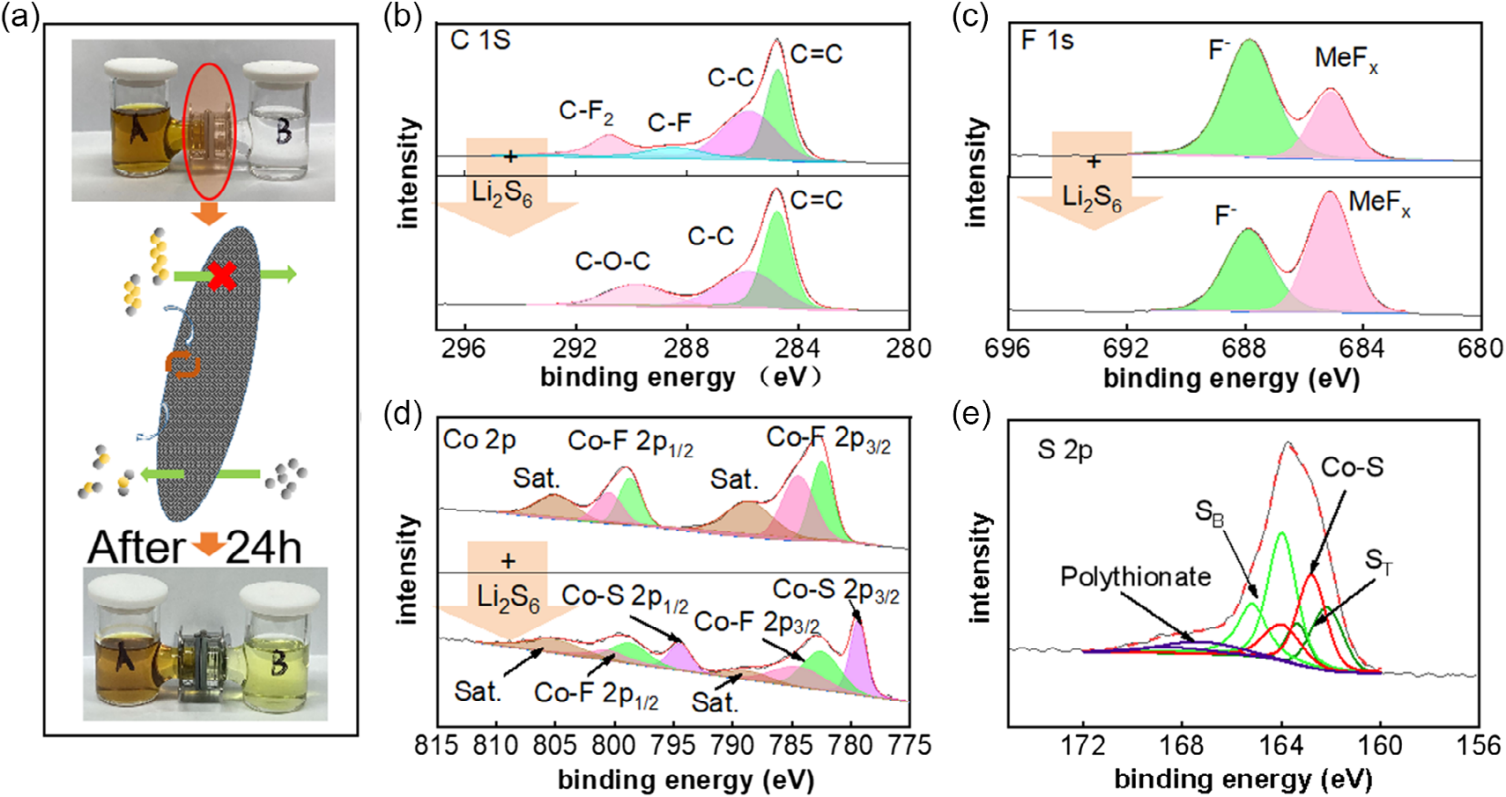
a) Schematic diagram of reduced shuttle of lithium polysulfide by modified separator and photos of Li_2_S_6_ solution before and after contacting with CoF_2_ for 24 h. b–e) XPS spectra: b) C 1s before and after adsorption of Li_2_S_6_; c) F 1s before and after adsorption of Li_2_S_6_; d) Co 2p before and after adsorption of Li_2_S_6_; and e) S 2p after adsorption of Li_2_S_6_.

As shown in **Figure** **5**, the visual experiment of polysulfides shuttling was designed to verify whether CoF_2_@C‐modified separator has a blocking effect on lithium polysulfide. The photograph in Figure 5a shows the change in the u‐tube solution after 24 h. The analysis in X‐ray photoelectron spectroscopy (XPS) spectra shows the interaction of Li_2_S_6_ and CoF_2_. The C 1s spectrum are shown in Figure 5b, presenting two distinct characteristic peaks located at 288.5 and 290.7 eV, corresponding to C–F and C–F_2_ in CoF_2_@C. After the adsorption of lithium polysulfide with CoF_2_, the characteristic peaks of 288.5 and 290.7 eV are significantly reduced. The high‐resolution F 1s spectrum of the modified separator exhibits two F 1s distinct characteristic peaks located at 684.9 and 687.9 eV. After the adsorption of lithium polysulfide with CoF_2_, the characteristic peaks of 684.9 eV are distinctly improved. This phenomenon indicates the production of more Li—F bonds. This can also explain the decrease in the percentage of peaks represented by the C—F bond in the C 1s. Figure 5d shows that the Co 2p spectrum of the CoF_2_ exhibits three doublets centered and the characteristic peaks Co 2p_3/2_ and Co 2p_1/2_ of CoF_2_ respectively. An adsorption peak representative of Co—S bond appears at 779.43 eV and 794.58 eV for CoF_2_–LiPS after the incorporation of lithium polysulfides. At the same time, the percentage of the peak area of the characteristic peaks of Co–F decreases obviously, which means that Co—S bond is formed in the reaction and indicates the strong interaction between CoF_2_ and Li_2_S_6_. In the S 2p core level of CoF_2_‐LiPS, there are two S 2p_3/2_ core levels with the ratio of 1:2 at 162.3 and 163.8 eV, respectively, corresponding to the terminal sulfur (S_T_) and bridging sulfur (S_B_). It could be found that after adsorption the Li_2_S_*x*_ (1 ≤ *x* ≤ 4) compounds are detected on the surface of CoF_2_ (binding energy of 164, 162−160 eV).^[^ ^39^ ^]^ Meanwhile two additional peaks corresponding to Co–S interaction are observed at 162.7 and 163.9 eV for CoF_2_–LiPS. All the above results indicate that the lithium polysulfide is adsorbed on the side of honeycomb structured CoF_2_@C and the shuttle effect is suppressed.

**Figure 6:**
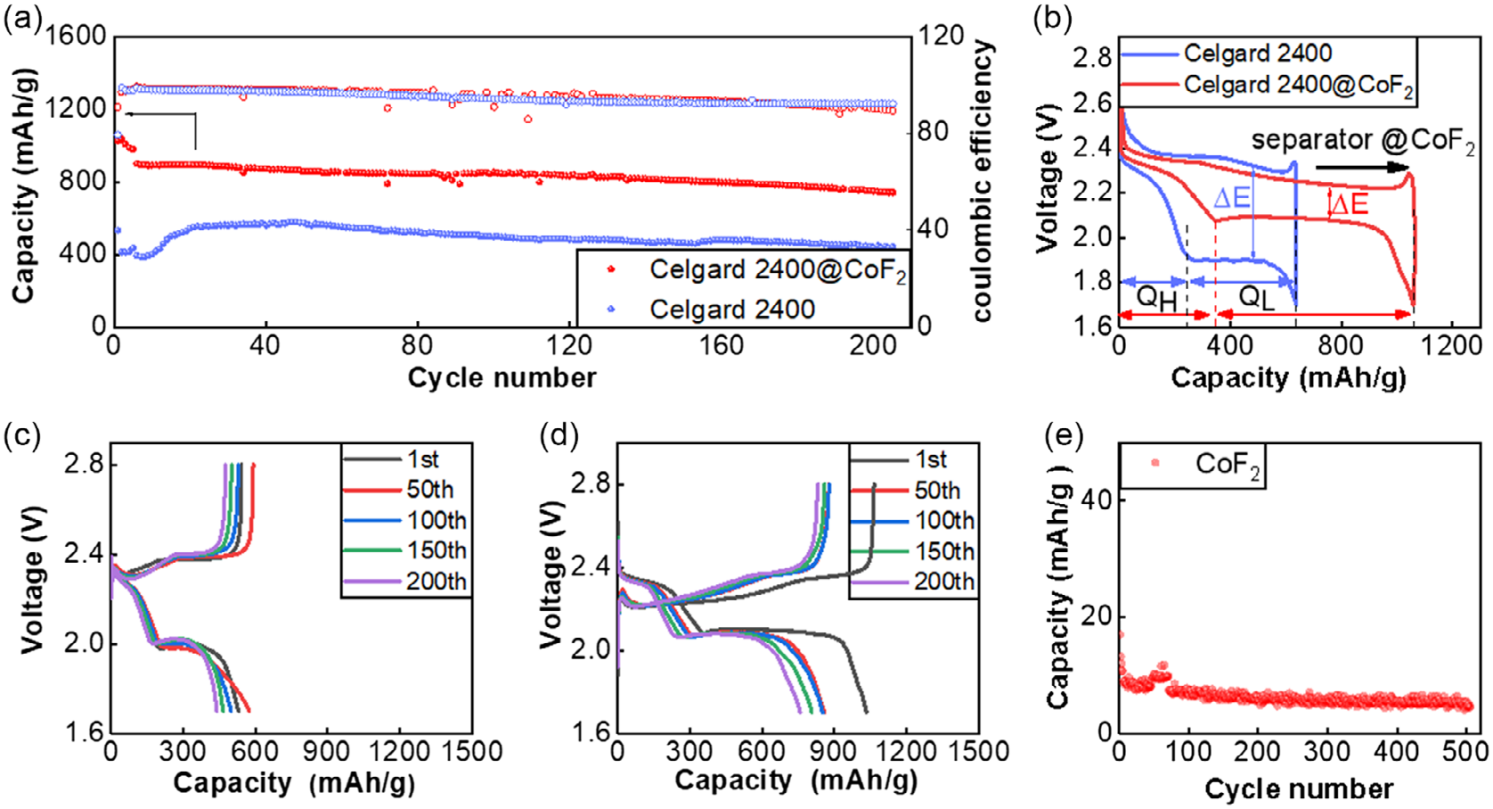
a) The long cycle at 0.2 C rate based on the mass of about 3.0 mg cm^−2^. b) Specific capacity–voltage graph of cell for one cycle; c,d) The cycle at 0.5 C rate based on the mass of about 4.0 mg cm^−2^. e) Capacity of CoF_2_ in the lithium–sulfur operating voltage platform.

Sulfur‐based composite electrodes with various sulfur loading (2.0–4.0 mg cm^−2^) are prepared to evaluate electrochemical performance. The cell with the modified separator first lap discharging reaches 899.5 mAh g^−1^. In comparison, the cell with PP separator first lap discharge with the same loading capacity was only 393.2 mAh g^−1^. The capacity of the cell with PP separator gradually increases during the first few turns of the cycle, indicating that the active material is not fully utilized. This is demonstrated that the separator modified with honeycomb CoF_2_@C facilitates the diffusive transport of lithium ions. From a complete charge and discharge cycle (as shown in **Figure** **6**b), the higher‐capacity utilization of the cell with modified separator is primarily reflected in the second platform of lithium–sulfur cells charge and discharge. This is due to the adsorption of lithium polysulfides on honeycomb CoF_2_, which inhibits the shuttle effect of lithium–sulfur cells, allowing the active substance to be fully utilized. Meanwhile, the activation energy required for Li_2_S oxidation during the charging process is lower than that in the cell with PP separator. Furthermore, Figure 6c,d shows that the polarization of the cells with the modified separator is significantly lower than that in the cell with PP separator. The difference in capacity is stark. In conjunction with the analysis in Figure 6e, the honeycomb CoF_2_@C material contributes almost no capacity in the same discharge–charge voltage range of lithium–sulfur chemistry, demonstrating that the honeycomb CoF_2_@C material only serves as an auxiliary in lithium–sulfur batteries and does not participate in the capacity contribution. As a result, after 200 discharge–charge cycles, the capacity of the cell with modified separator capacity still provides 740.4 mAh g^−1^, while the cell with PP separator demonstrates a specific capacity of 441 mAh g^−1^. The cell with the modified separator capacity retention rate reaches 82.3%, while the cell with PP separator battery capacity retention rate is only 76.2%.

**Figure 7:**
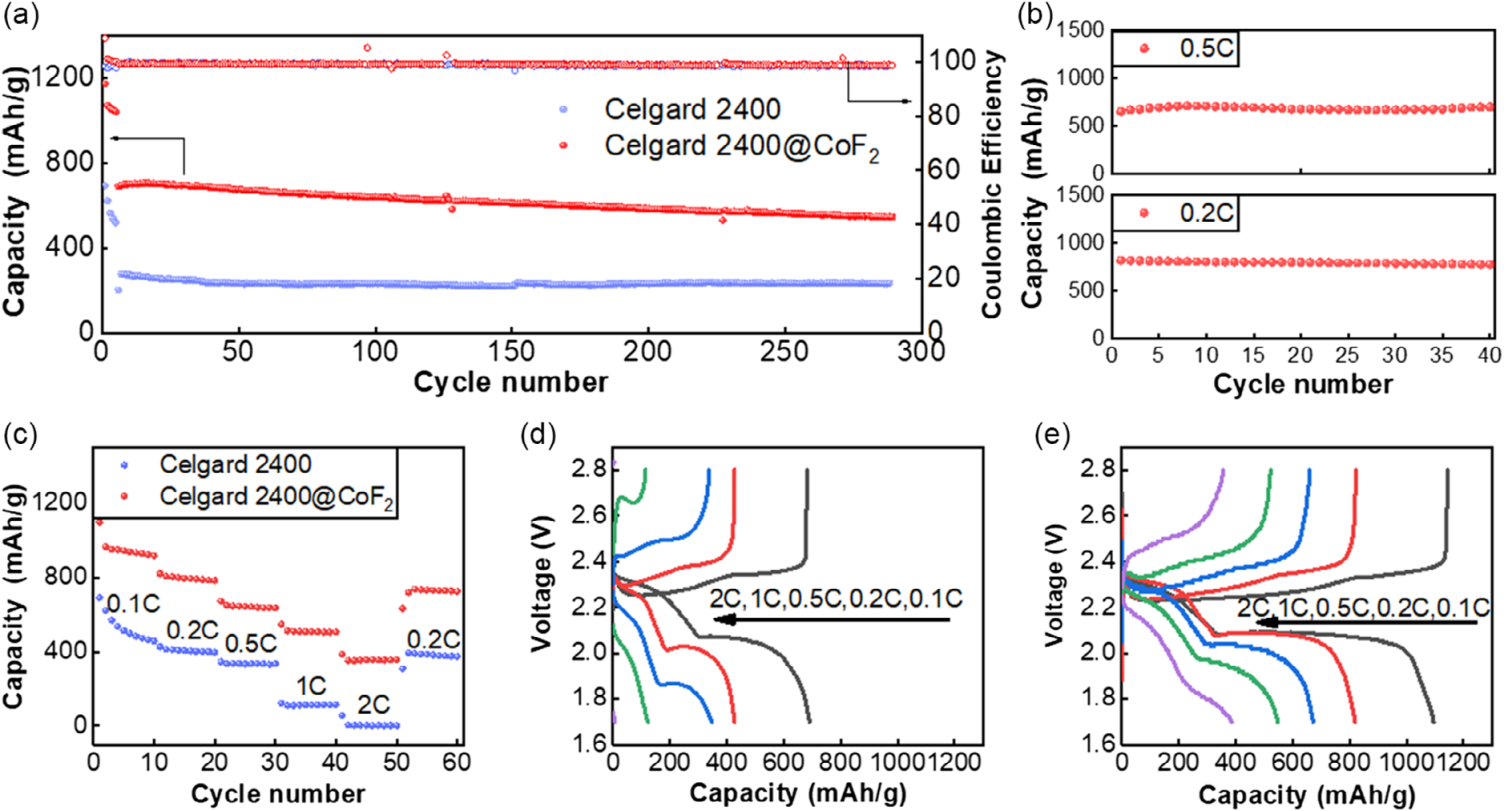
a) The long cycle at 1 C rate based on the mass loading of 2.0 mg cm^−2^. b) The cycle at 0.2 and 0.5 C rate based on the mass loading of 4.0 mg cm^−2^. c) The rate performance at various rates. d,e) Discharge–charge curves of the cell with or without modified separator at different C rates.

The galvanostatic charge–discharge tests at 1 C were further investigated. At a sulfur loading of 2.0 mg and an E/S of 15 μL mg^−1^, the first cycle of charging and discharging cell capacity is 687.8 mAh g^−1^ at 1 C (as shown in **Figure** **7**a). Meanwhile, the capacity of the cell with PP separator is only 274.3 mAh g^−1^. Such a contrast is striking. After 300 cycles, the Li–S cells with CoF_2_@C‐modified separator still deliver a discharge capacity of 542.4 mAh g^−1^ with a capacity retention of 77.2%. As shown in Figure 7b, with higher sulfur loading of 4 mg cm^−2^ and the E/S of 10 μL mg^−1^, the cells could continue to function and maintain a capacity of about 818.1 mAh g^−1^ at 0.2 C and 690 mAh g^−1^ at 0.5 C. Concerning the rate performance of the cells, CoF_2_@C‐modified separator promotes discharge capacities of 1096, 819.5, 672.2, 547.2, 386.3, and 737.1 mAh g^−1^ at various rates from 0.1–2 C, which are superior than those of the cell with PP separator. The second discharge plateau capacity of the cell is significantly higher than that of the control group, according to the charging and discharging curves. Additionally, the cell with modified separator can continue to operate steadily when the discharge rate is 2 C, whereas the cell with PP separator capacity is virtually nonexistent. When the discharge rate is switched back to 0.2 C rate, the capacity is recovered to 718.5 mAh g^−1^, suggesting the good stability and reversibility of the cell with CoF_2_@C‐modified separator in the discharge–charge process. All the above results present that the separator modified with honeycomb structured CoF_2_@C in Li–S cell improves all aspects of cell performance.

**Figure 8:**
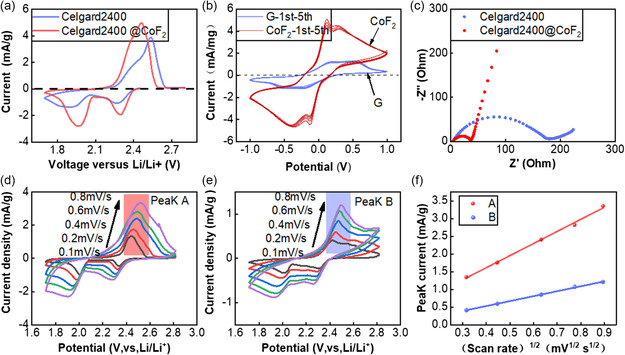
a) The CV curves of the cells with or without modified separator. b) CV curves of the symmetric cells with graphite and CoF_2_. c) EIS spectra of cells with with or without modified separator. d) CV curves of the cells with modified separator at various scan rates. e) CV curves of the cells without modified separator at various scan rates. f) Plots of peak current (*I*_p_) for anodic oxidation process with the square root of the scan rate (mV^1/2^).

In the cyclic voltammetry (CV) tests, **Figure** **8**a shows that the cell with the modified separator has a higher response current during the charging and discharging process. It can also be seen that the three peaks of the charging and discharging process of the cell with the modified separator are 1.972, 2.304, and 2.464 V, respectively, which has a lower redox potential than the cell with PP separator, implying that the honeycomb CoF_2_@C can reduce the activation energy of the reaction and facilitate the transformation of lithium polysulfides. Then we have performed an electrochemical analysis of the catalytic effect of honeycomb CoF_2_@C on lithium polysulfide.^[^ ^40^ ^]^ A certain amount of honeycomb CoF_2_@C or graphite are coated on the carbon cloth and then the produced electrodes are assembled into a symmetrical cell with Li_2_S_6_ solution. Cell charge and discharge tests were conducted in the voltage range of −1 to 1 V. CoF_2_@C demonstrates a larger response current, as shown in Figure 8b. The clear distinction suggests that the honeycomb CoF_2_@C has a far stronger catalytic impact on lithium polysulfide than graphite. This is in line with the results of CV. According to electrochemical impedance spectroscopy (EIS) tests, the cell with modified separator electrical resistance is significantly lower than that of the cell with PP separator simply. The small values of the impedance indicate the rapid lithium‐ion transport in the cell with CoF_2_@C‐modified separator. Meanwhile, CV tests under different sweep rates were carried out. As presented in Figure 8d,e, the lithium‐ion diffusion coefficients (D_Li+_) of the lithium–sulfur battery cathodes were studied.^[^ ^41^ ^]^ The relationship between the peak current density and scanning rate can reflect the value of D_Li+_. The calculation process is based on the Randles–Sevick equation given below

(1) $$I_{p}=2.69\times 10^{5}n^{\frac{3}{2}}\text{ A}D^{\frac{1}{2}}\text{ C}v^{\frac{1}{2}}$$

where *I*_p_ corresponds to the peak current (A). n is the number of electrons transferred in the reaction. *A* is the electrode area (cm^−2^). *D* is the diffusion coefficient of lithium ion. *C* represents the lithium‐ion concentration (molL^−1^) and *v* is the scan rate (V s^−1^). First, each current value of the cell with the modified separator (peak A) is significantly larger than the cell with PP separator (peak B) at the same sulfur loading. The linear fitting curves of the normalized peak current to the square root of the scanning rate and the calculated *D* value are shown in Figure 8f. Obviously, the slope of peak A is steeper than peak B, indicating a higher diffusion coefficient of lithium ion. In summary, the honeycomb CoF_2_@C‐modified separator significantly improves the kinetic of sulfur redox reaction.

**Figure 9:**
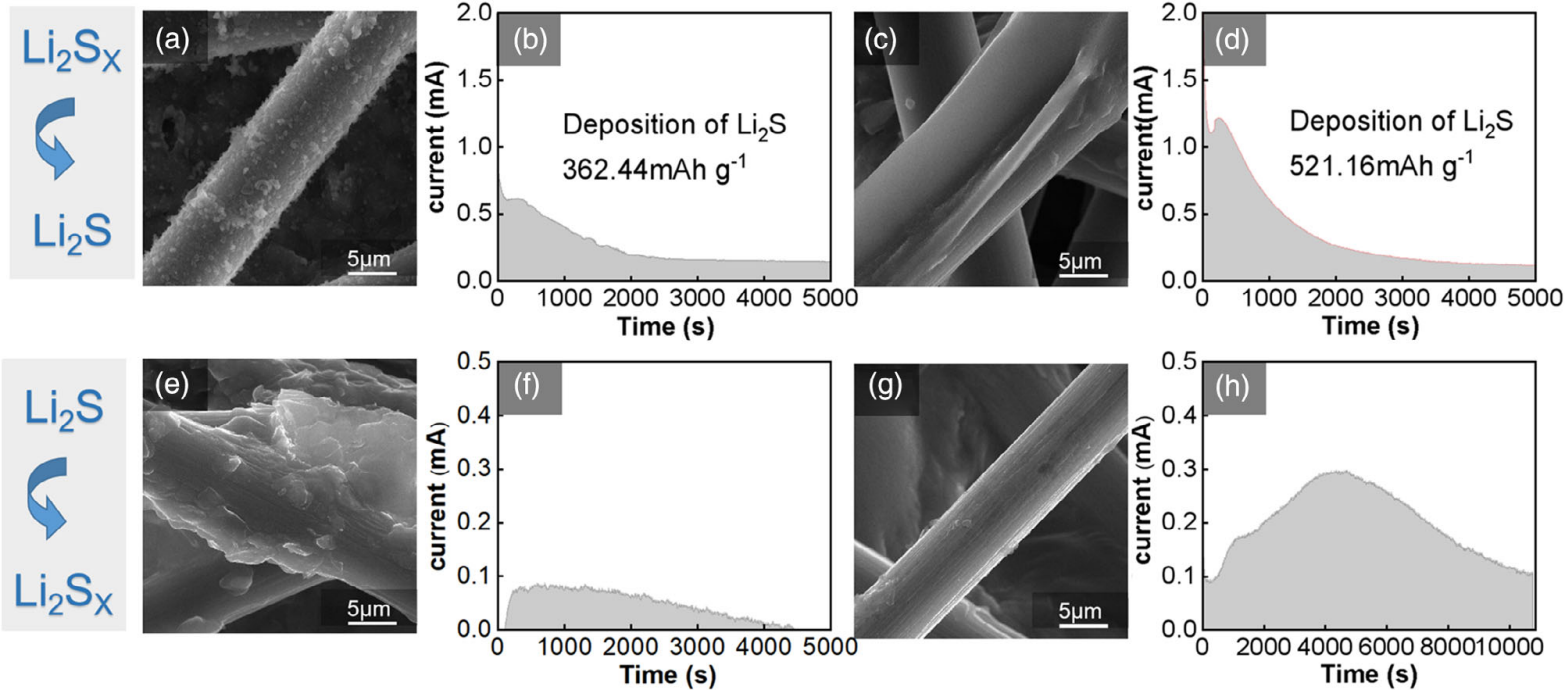
a) The morphology of Li_2_S nucleation on cells without modified separator. b) Potentiostatic discharge profiles of Li_2_S_8_ solutions on cells without modified separator at 2.05 V. c) The morphology of Li_2_S nucleation on cells with modified separator. d) Potentiostatic discharge profiles of Li_2_S_8_ solutions on cells with modified separator at 2.05 V. e) Morphology of Li_2_S dissolution on cells without modified separator. f) Potentiostatic charge profiles of cells without modified separator at 2.3 V. g) Morphology of Li_2_S dissolution on cells with modified separator. h) Potentiostatic charge profiles of cells with modified separator at 2.3 V.

The oxidation and reduction of Li_2_S was investigated through potentiostatic charging experiments (as shown in **Figure** **9**). The decomposition capacity by the quantity of electric charge is much higher for CoF_2_@C‐modified separator than the regular separator, suggesting the effective oxidation of Li_2_S on carbon paper surface with the modified separator. Meanwhile, SEM images show that the precipitated Li_2_S still exists on carbon paper surface with a regular separator surface after oxide reaction (Figure 9e), while it almost disappears on carbon paper surface with a modified separator (Figure 9h), suggesting the facilitated decomposition of Li_2_S on carbon paper surface with the modified separator during the charging process. The results clearly demonstrate that CoF_2_@C promotes the precipitation and decomposition of Li_2_S.

**Figure 10:**
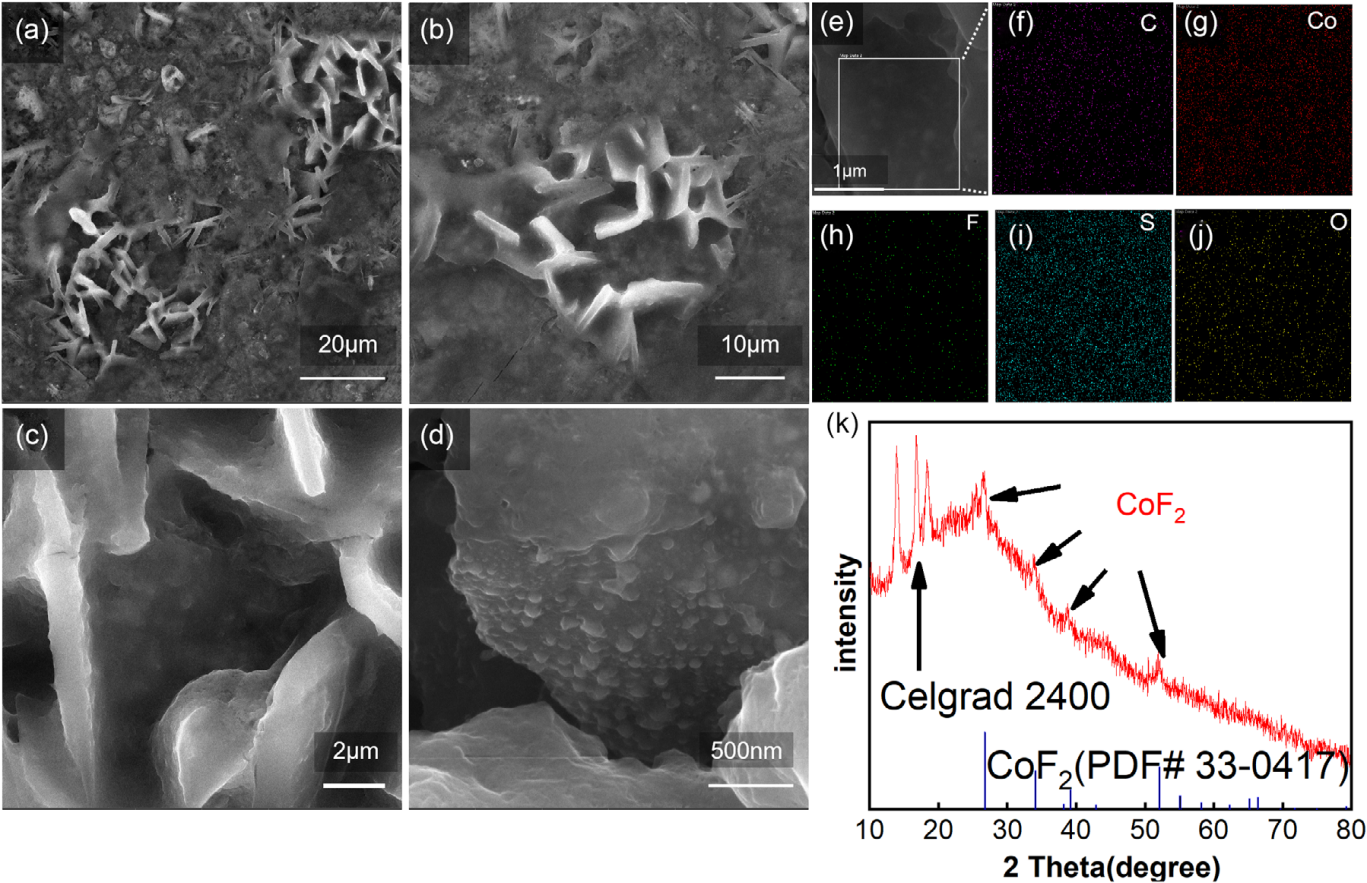
a–d) SEM images of CoF_2_@C on modified separator (after 300 cycles). e–j) EDS images: e) their overlapped maps; f) the carbon; g) the cobalt; h) the fluorine; i) the sulfur; j) the oxygen; k) XRD pattern of CoF_2_ in modified separator (after 200 cycles).

**Figure 11:**
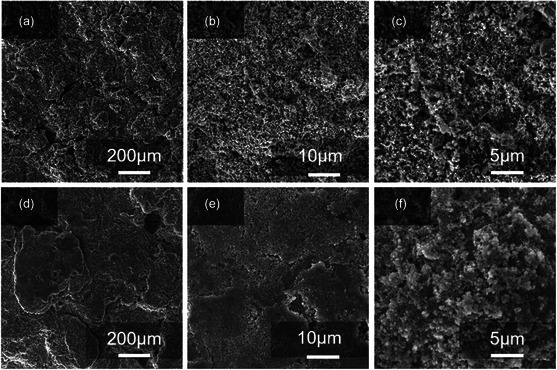
a–c) SEM images of the cycled cathode in the cell with modified separator (after 200 cycles). d–f) SEM images of cycled cathode in the cell with PP separator (after 200 cycles).

**Figure 12:**
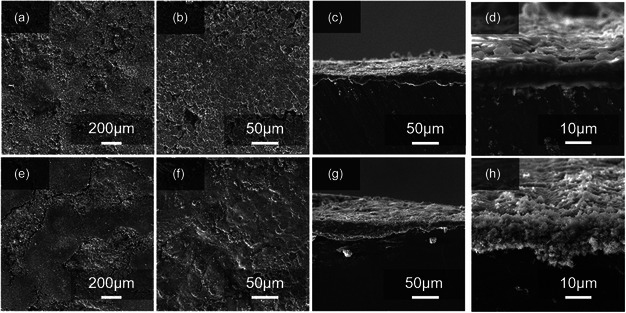
a–d) SEM images of the front and side views of cycled anode in the cell with modified separator (after 200 cycles). e–h) SEM images of the front and side views of cycled anode in the cell with PP separator (after 200 cycles).

The morphologies of cycled electrodes and modified separator were studied. We have analyzed the SEM characterization of the cycled modified separator (as shown in **Figure** **10**). The CoF_2_@C of the modified separator still has good morphological retention after 300 cycles in Figure 10a‐d. More lithium polysulfide deposited especially on the surface of the carbon wall due to the adsorption of polysulfide on the CoF_2_. Meanwhile, we analyzed the XRD of the cycled modified separator. XRD result shows that CoF_2_ still has good stability after 200 cycles in Figure 10k. CoF_2_ phase is verified by the four typical peaks at 26.7°, 34.0°, 39.1°, and 52.0°, which agree with PDF card of CoF_2_ (No. 33‐0417). The first three peaks are from the cell separator.^[^ ^42^ ^]^ The results show that CoF_2_ still has good stability of morphological retention and phase after long‐term cycling. **Figure** **11** shows the surface of cathodes in the cells after 200 cycles, respectively. As shown in Figures 11a–c, the sulfur cathode in the modified cell has a clean surface, indicating that the shuttle of the polysulfides is alleviated in cycles, which is favorable to reduce the unfavorable precipitation of lithium polysulfide. This should be attributed to the insertion of the honeycomb structured CoF_2_@C‐modifed separator. In contrast, the visible species precipitation from lithium polysulfides is found on the conventional cells (Figure 11d–f). The SEM images of the front and side views of the cycled lithium foil are demonstrated in **Figure** **12**, showing the extent of lithium metal corrosion. The front side of the lithium foil sheet in Figure 12a,b is more compact and smoother with the modified separator. While lithium foils without the CoF_2_@C‐modified separator (as shown in Figure 12e,f) show uneven lithium deposition with many patches and pores on the surface, this indicates that the modified separator can effectively reduce the polysulfide shuttle in the battery reaction, reducing the reduction of lithium polysulfide on the Li surface and causing the smooth and dense surface on the cycled Li metal. This conclusion is consistent with the above results from the SEM images of cycled cathodes. From the side view of the lithium foil after cycling, the formed lithium dendrite (as shown in Figure 12c,d) is much smaller than that from Figure 12g,h. A severe lithium dendrite phenomenon can lead to short circuiting of the battery by puncturing the separator during charging and discharging. This apparent difference indicates that the modified separator is very effective in suppressing the lithium dendritic growth. The separator modified by the honeycomb structure CoF_2_@C shows protective properties of the anode and catalytic performance of accelerating the Li–S conversion reaction kinetics.

## 3 Conclusion

We have developed a honeycomb structured CoF_2_@C with a 3D conductive network to modify the separator in Li–S battery. Honeycomb structured CoF_2_@C‐modified separator not only reduces the electrochemical impedance of the cell, but also provides excellent adsorption and electrocatalytic activity toward soluble intermediate polysulfide species during battery storage and cycling processes. In the meantime, the honeycomb structured CoF_2_@C‐modified separator with inner pore spaces achieves rapid and uniform lithium‐ion transport. Due to the combined effects, the Li–S cells with a modified separator demonstrate higher rate capability and cycle stability. Furthermore, a honeycomb structured CoF_2_@C‐modified separator can promote high electrochemical performance under high sulfur loading conditions and low E/S ratios. In contrast to the complex synthesis of nanocomposite materials as hosts, we use a wide and inexpensive source of metal fluoride materials produced by a simple method. It is very significant to estimate prospectively practical application by fitting Li–S pouch cells with a high S loading and low E/S ratios.

## 4 Experimental Section

4.1

4.1.1

##### Preparation of Graphite/Sulfur Composites

The graphite and sulfur were added to the agate mortar at a mass ratio of 1:3, and absolute ethyl alcohol was added for homogeneous mixing. After fully grinding and mixing, the graphite/sulfur composites were washed three times with deionized water and ultrasonic processing. Finally, this powdered material was drying by a drying machine at 45 °C for 24 h.

##### Preparation of Honeycomb CoF_2_@C

The 0.5 g cobalt nitrate and 0.5 g poly(vinylpyrrolidone) (PVP) were added to the beaker, with 100 mL deionized water for homogeneous mixing. The solution was completely dried to form a PVP–cobalt nitrate composite cluster. The cluster was carbonized and fluoridized for 6 h at 750 °C under argon‐protected atmosphere and 3 h at 280 °C under argon and NF_3_ atmosphere, respectively. Finally, the honeycomb‐structured CoF_2_@C powder, PVDF, and super‐P (quality ratio is 8:1:1) were dispersed in a separator by the coating process method.

##### Adsorption Experiment of Lithium

Li_2_S_6_ solution (50 × 10^−3^ m) was prepared by mixing Li_2_S and sulfur powder(molar ratio, 1:5) into DOL/DME solvent (v/v, 1:1) and magnetic stirring at 65 °C for 72 h until dissolved completely. 50 mg amount of graphite powder and CoF_2_@C powder was added to Li_2_S_6_ solution (5 × 10^−3^ m) diluted by DOL/DME solvent. Adsorption was performed for 24 h, followed by UV testing. All operations were completed in an Ar‐filled glovebox.

##### Electrochemical Measurement

The working electrodes consisted of 80 wt% as‐prepared composite, 10 wt% super P, and 10 wt% polyvinyldifluoride (PVDF). The powder mixed was dispersed in *N*‐methyl‐pyrrolidone (NMP). The slurry was stirred in agate mortar, coated onto Al foil, and then dried at 40 °C overnight. The modified Celgard 2400 was coated by a layer of slurry which consisted of 80 wt% honeycomb CoF_2_@C, 10 wt% super P, and 10 wt% PVDF. Finally, the working electrodes were punched into disks with a diameter of 12 mm, and the Celgard 2400@CoF_2_@C was punched into disks with a diameter of 19 mm. The sulfur content of low loading electrodes was 1.5–2.5 mg cm^−2^, and high‐areal sulfur loading electrode of 3.0–5.0 mg cm^−2^ was produced. The electrolyte/sulfur ratio was about 10–20 μL mg^−1^ for the tests. The electrolyte consisted of 1.0 m LiTFSI with 2 wt% LiNO_3_ in 1,2‐dimethoxyethance (DME) and 1,3‐dioxolane (DOL) (v/v, 1:1). The galvanostatic discharge/charge tests were carried out with LAND‐CT3001A instruments in the potential range of 1.7–2.8 V. CV, data management, and electrochemical impedance analysis were performed using Gammry workstation (Reference 600+, Gamry Instruments, USA).

##### DFT

First‐principle calculations based on DFT method were performed using the Vienna Ab initio Simulation Package (VASP). The cutoff energy for the planewave expansion of the PAW basis set was set to be 450 eV, and 3 × 3 × 1 Γ‐centered *k*‐point grids were used for Brillouin zone integrations. The exchange‐correlation functional with a Gaussian smearing width term of 0.05 eV was used.

##### Structure Characterization

Micrographs was conducted with SEM (TESCAN MIRA) at an accelerating voltage of 5 kV. BF‐ and HAADF‐TEM images were conducted with a transmission electron microscope (Talos‐S) at an accelerating voltage of 20–200 kV. The crystal structure of the prepared materials was studied using an X‐ray diffractometer (Empyrean 2) within a 2*θ* range of 10°–80°. The surface composition of the CoF_2_@C was investigated using XPS analysis recorded on Thermo Scientific ESCALAB250Xi with Al Kα radiation (hν = 1486.6 eV). The remaining polysulfides in supernatant after adsorption were measured using UV–vis (UV2600).

## Conflict of Interest

The authors declare no conflict of interest.

## Data Availability

The data that support the findings of this study are available from the corresponding author upon reasonable request.
